# Recurrent Flank Hernia Repair After Penetrating Abdominal Wall Trauma

**DOI:** 10.7759/cureus.114084

**Published:** 2026-08-06

**Authors:** Stephanie Nguyen, Yannis Karamitas, Alexander Vu, Ryan Robalino, Omkaar Jaikaran, Derek Lim, Prashant Sinha

**Affiliations:** 1 College of Osteopathic Medicine, Touro College of Osteopathic Medicine, Las Vegas, USA; 2 General Surgery, Maimonides Medical Center, Brooklyn, USA; 3 Minimally Invasive Surgery, NYU Langone Health, Brooklyn, USA; 4 Transplant Surgery, NYU Langone Health, New York, USA; 5 Surgery, South Shore University Hospital, Bay Shore, USA; 6 Surgery, Atlantic Health System, Pompton Plains, USA; 7 Surgery, Newark Beth Israel Medical Center, Newark, USA

**Keywords:** abdominal wall, abdominal wall surgery, delayed reconstruction, flank hernia, penetrating abdominal trauma

## Abstract

Traumatic abdominal flank hernias are rare and challenging but more common than previously recognized. There is no consensus on the management of traumatic flank hernias. The majority of studies have been conducted in the setting of blunt trauma; however, there is a paucity of literature on penetrating trauma. We present the case of a man in his thirties with a persistent left flank hernia after sustaining penetrating abdominal wall trauma from a tree branch two years earlier and undergoing multiple subsequent reconstructive surgeries, including both open and minimally invasive approaches. Flank hernia repair is most commonly performed with mesh and can be accomplished using either an open or minimally invasive approach, either at the time of trauma or in a delayed fashion. In selected patients, definitive repair of traumatic abdominal wall defects may be delayed until acute injuries and contamination have resolved. In this patient, intra-abdominal injuries required immediate operative management, whereas definitive reconstruction of the subsequent symptomatic flank hernia was performed in a delayed fashion.

## Introduction

Flank hernias are rare abdominal wall hernias that occur lateral to the rectus sheath, extending 3 cm above and below the umbilicus, as defined by the European Hernia Society [1]. These hernias are further classified as primary or secondary, the latter of which includes traumatic etiologies.

Traumatic abdominal wall defects are uncommon injuries and are most frequently reported following blunt trauma. Penetrating trauma causing extensive flank muscular and fascial disruption with subsequent flank herniation is less well described. A study by Harrell et al. reported 281 cases of traumatic abdominal wall hernias (TAWHs) from 2012 to 2018 [2]. Although these injuries are more common than previously recognized, there is no consensus on the surgical management of traumatic flank hernias.

The present case differs from previously reported traumatic flank hernias because it describes the complete operative course of a patient who sustained a motor vehicle collision involving a tree trunk, resulting in impalement, initial reconstruction, and subsequent robotic-assisted repair of a recurrent incisional hernia on two occasions. To our knowledge, there is a paucity of literature on the management of traumatic flank hernias, and our case provides insights into the operative decision-making and robotic techniques used in this complex reconstruction at an academic teaching hospital.

This manuscript provides an overview of the literature on traumatic flank hernias, including the pathophysiology, diagnosis, and management of these injuries. A PubMed search was performed using the strategy ((“Hernia, Abdominal”[MeSH]) OR “hernia”) AND “flank” without additional filters on January 5, 2025. This search yielded 299 articles, which were manually screened by the authors. Traumatic lateral abdominal wall defects involving the flank or lateral abdominal wall were included, with lumbar hernias identified separately where applicable. Non-abdominal, inguinal, non-traumatic primary hernias, postoperative incisional hernias unrelated to traumatic abdominal wall disruption, and non-English-language literature were excluded. A total of 17 studies met the inclusion criteria and were included in the literature review.

Given the limited data on penetrating traumatic flank hernias, a second PubMed search was performed using the strategy ((“Hernia, Abdominal”[MeSH]) OR “hernia”) AND “flank” AND (“trauma” OR “traumatic”) AND “penetrating” on January 5, 2025. This search identified 98 articles, which were again manually screened by the authors using the same inclusion and exclusion criteria as the first search. Only three studies met the inclusion criteria, none of which overlapped with the 17 studies identified in the first search, highlighting the marked paucity of literature describing penetrating traumatic flank hernias.

There are no standardized guidelines addressing the management of traumatic flank hernias, particularly those resulting from penetrating trauma. The literature supports open repair, laparoscopic approaches (transabdominal preperitoneal (TAPP) or total extraperitoneal (TEP)), and hybrid approaches for flank hernia management [3]. Studies suggest lower recurrence and complication rates with laparoscopic repair compared with open repair, with recurrence rates ranging from 0 to 2.9% for laparoscopic repairs versus 0 to 15.9% for open repairs [2]. Regardless of the surgical approach, there is general consensus that mesh should be used for traumatic flank hernia repair [4].

Multiple approaches exist for flank hernia repair, including open, laparoscopic, and hybrid minimally invasive techniques. These involve varying methods of mesh placement (TAPP, TEP, or intraperitoneal) and fixation (tacks, sutures, or bone anchors). The primary challenges are achieving secure mesh fixation, effective defect closure, and restoration of abdominal wall function while avoiding nerve injury or entrapment.

This article was previously presented as a meeting abstract at the 2021 Society of American Gastrointestinal and Endoscopic Surgeons (SAGES) Annual Meeting on August 31, 2021.

## Case presentation

**Figure 1:**
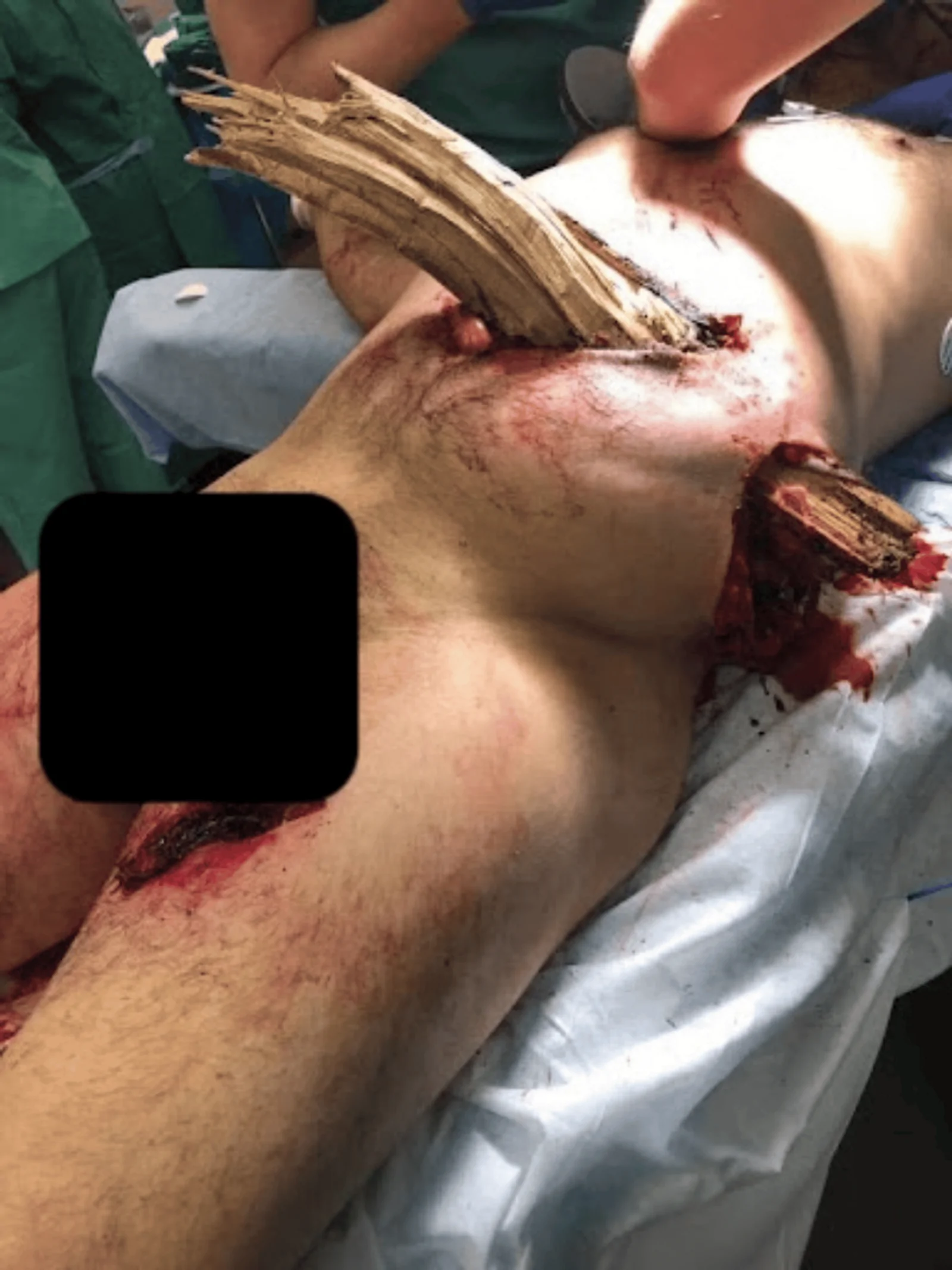
Patient presenting with Level I trauma following tree branch impalement to the left lower quadrant, with a penile degloving injury and a laceration of the left anterior thigh

We present the case of a man in his thirties with no significant past medical history who was brought to the emergency department by ambulance after a motor vehicle collision with a tree, followed by impalement by a tree branch that penetrated his car and entered his left lower quadrant (Figure 1). He underwent removal of the penetrating object, debridement, colon anastomosis, and wound closure. Seven months after discharge, the patient underwent left flank hernia repair with mesh. Subsequently, he developed recurrent left flank hernias and underwent robotic incisional hernia repair with mesh on two occasions.

When the patient initially presented to the emergency department as a Level 1 trauma, the skin bridge between the entry and exit wounds created by the branch was divided with a scalpel, and the subcutaneous tissue was divided using electrocautery. This allowed for safe extraction of the tree branch, leaving a 20 cm defect extending from the left rectus sheath to the iliac spine. At the time of extraction, no active bleeding was observed, allowing copious irrigation of the wound to minimize contamination.

An intra-abdominal examination revealed a transection injury of the sigmoid colon with an associated mesenteric laceration. The injured segment was resected, and the mesentery was clamped and ligated, leaving the bowel in discontinuity. An injury to a sigmoid artery was also identified and ligated.

The musculature of the left abdomen was completely disrupted, presenting both the potential for loss of function and a significant challenge to achieving primary closure. Nonviable tissue was sharply debrided, and following irrigation, an ABThera VAC was applied to facilitate wound management and drainage. The patient remained intubated and was transferred to the surgical intensive care unit in stable condition.

Due to gross contamination of the patient's wound, a decision was made to return to the operating room (OR) for washout and additional debridement. The abdominal cavity was irrigated copiously with warm saline in an attempt to remove as much gravel as possible. The abdominal wall was again debrided to healthy, bleeding tissue, and hemostasis was confirmed. The omentum was then positioned to cover the small bowel, and the ABThera VAC was replaced.

Two days later, the patient returned to the OR for colon anastomosis. The left colon was mobilized by releasing the peritoneal reflection, and a stapled side-to-side anastomosis was performed. The common enterotomy was then hand-sewn in two layers, the abdomen was irrigated, and a new ABThera VAC was applied.

Two days after the colon anastomosis, the patient returned to the OR for wound closure and soft tissue coverage using Strattice underlay mesh. The fascia could not be closed primarily, but the skin was approximated with minimal mobilization. A 20×40 cm piece of Strattice was placed within the peritoneal cavity as a bridging underlay and anchored circumferentially to the iliac crest periosteum and through the full thickness of the muscle and fascia. Interrupted sutures were placed several centimeters from the fascial edge to provide adequate underlay overlap; however, the mesh overlap distance was not documented in the operative report.

The portion of the muscle and fascia in the midline that could be closed primarily was approximated with figure-of-eight sutures. The skin and subcutaneous tissues were elevated circumferentially from the fascia using electrocautery to allow for primary closure. All necrotic skin edges were trimmed back to healthy, bleeding tissue. The skin was closed, and an ABThera VAC dressing was applied.

The patient recovered well after placement of the bridging Strattice underlay mesh but subsequently developed a symptomatic left flank hernia seven months later. He returned to the OR for open hernia repair with onlay mesh. A transverse incision was made through the previous scar, and the subcutaneous tissue was divided. Skin and subcutaneous tissue flaps were elevated circumferentially off the muscle fascia to the level of the costal margin, the anterior superior iliac spine (ASIS), posterior to the hernia, and medially to the midline. The incorporated Strattice mesh appeared healthy, and the defect was located horizontally in the left flank.

By freeing the muscle from the adherent subcutaneous scar, the muscle and fascial edges could be mobilized inferiorly and superiorly to meet. The fascia was closed horizontally by first placing all sutures, elevating the fascia to protect the bowel, and tying the sutures sequentially. Airway pressures were not affected by this maneuver. Hemostasis was achieved, and the wound was copiously irrigated with saline.

**Figure 2:**
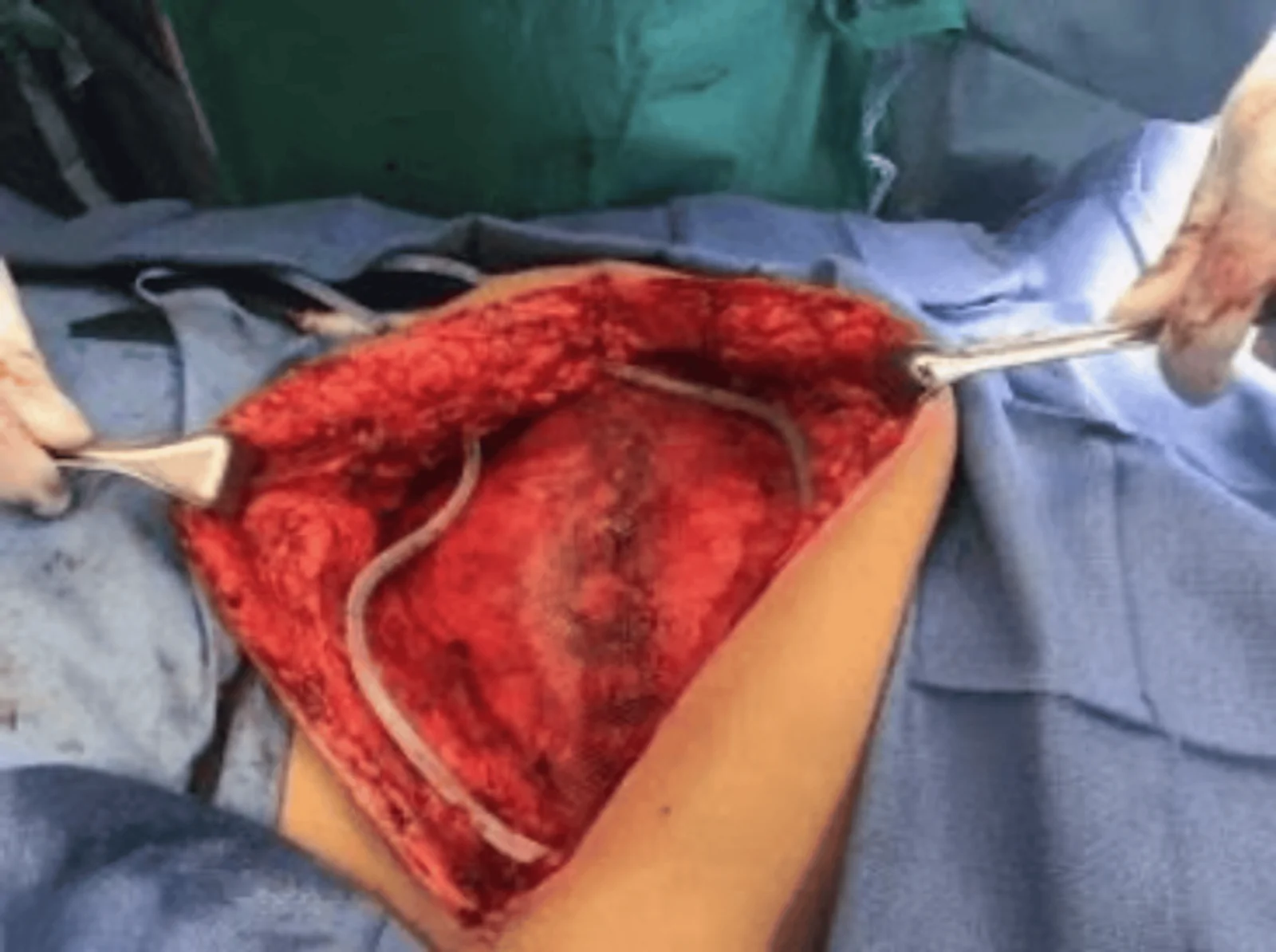
Intraoperative image showing open left flank hernia repair with a secured 30×30 cm Parietex mesh overlay and two Blake drains

A 30×30 cm Parietex mesh was trimmed for use as an overlay on the exposed fascia. It was secured in place with multiple #1 Maxon sutures, as shown in Figure 2. Excess skin and subcutaneous tissue were trimmed with a knife and electrocautery. The subcutaneous tissues were closed with Vicryl, and the skin was closed with staples. A Prevena dressing and an abdominal binder were applied.

Four months after the previous repair, including two formal hernia repairs with Strattice mesh followed by suture buttressing with external Parietex mesh, the patient presented with an enlarging left flank bulge. He provided informed consent for robotic incisional flank hernia repair with mesh.

Ports were placed at the left subcostal midclavicular line, two at the midline, and a fourth lower port just left of the midline. The robot was docked, and the instruments were inserted. Extensive lysis of the scarred omentum, requiring approximately 45 minutes, was performed to expose the hernia defect. After lysis of adhesions and medialization of the left colon, a preperitoneal flap was attempted but later abandoned because of scarring and insufficient peritoneum. Direct closure of the defect was deemed infeasible because of excessive tension.

**Figure 3:**
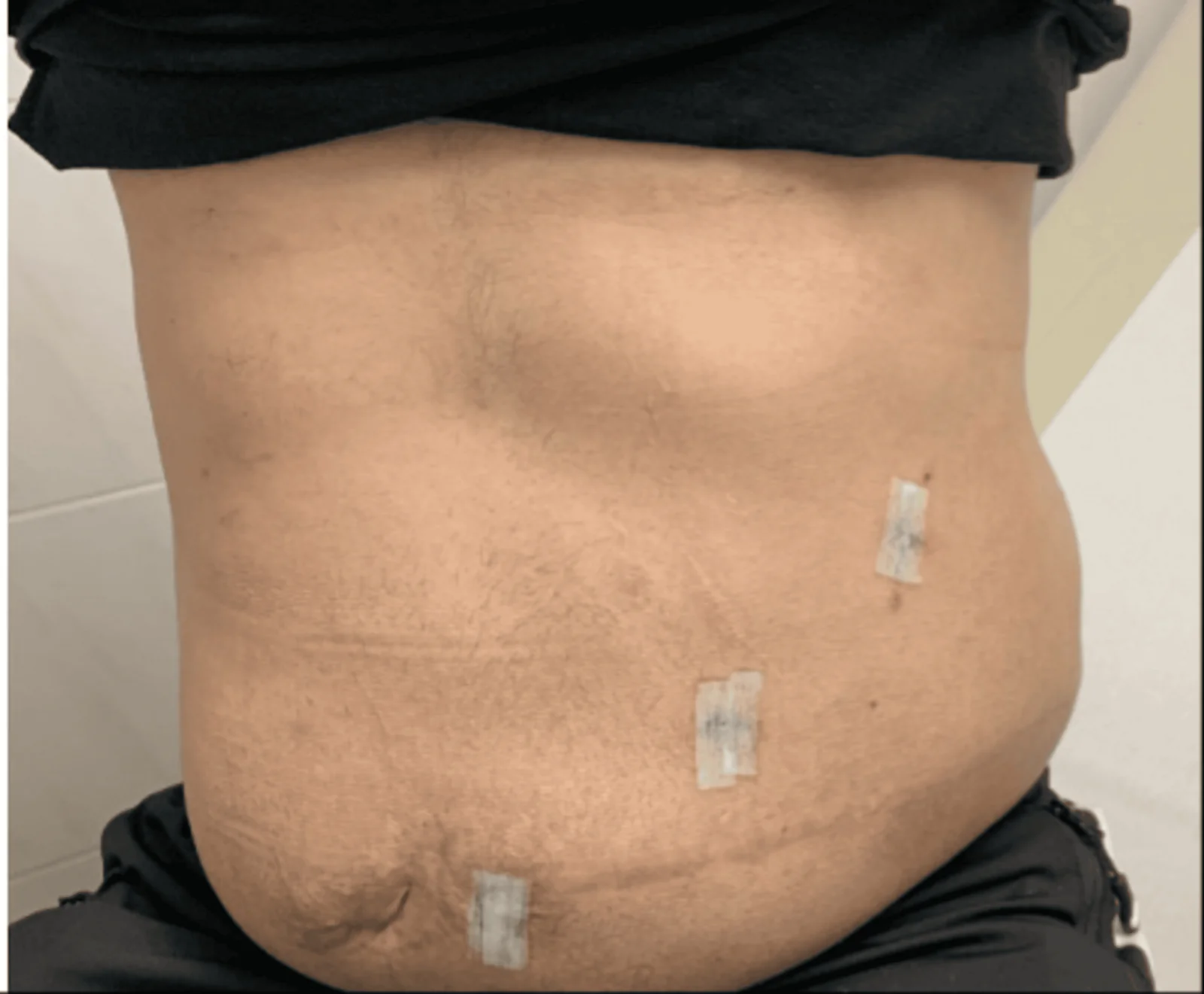
Patient cosmesis after the first robotic hernia repair with mesh placement

To address the partial loss of abdominal musculature with a fascial defect that could not be closed, as well as the diastasis of the remaining musculature, the diastatic area was plicated, incorporating the fascial edges of the defect to eliminate any interparietal defect between muscle bodies and retension the fascia to create a continuous line of tension across the abdominal wall. The eventration was sequentially plicated with a series of 0 V-Loc sutures at 8 mmHg until the defect was approximately flattened from the costal margin to the iliac crest. A 20×25 cm Parietex mesh was then inserted, positioned over the defect, and secured with four running 2-0 V-Loc suture lines along the cranial, caudal, medial, and lateral margins. AbsorbaTack was used to secure several edges and the center of the mesh to promote early scarring and integration with the plicated musculature. Effectively, the remaining layers of the posttraumatic abdominal wall were fused into a continuous layer in the area of the defect. The patient returned for follow-up, and the cosmetic result at this stage of the delayed repair is shown in Figure 3.

The patient's surgical course was further complicated by recurrent herniation and a new defect in the space between the flank and the left rectus muscle. Four months later, two midline incisional hernias and an area of diastasis along the transverse left lower abdominal incision were identified. The patient provided informed consent for robotic incisional hernia repair with mesh. Ports were placed along the right subcostal midclavicular line and between the costal margin and the anterior superior iliac spine, after which the robot was docked. Loose adhesions involving the intestine and omentum were identified along the abdominal wall and were lysed using sharp dissection, while omentum and fat were reduced from two 3 cm incisional defects in the left lower midline within the symptomatic diastasis field. The area of diastasis, measuring approximately 20×15 cm, began at the umbilicus, extended along the left abdomen where the rectus muscle had been destroyed, and terminated at the site of the previous mesh repair of the left flank hernia. A small diastasis within the superior portion of the previous left flank hernia repair was also identified.

The midline incisional defect was closed with 0 V-Loc sutures, followed by plication of the left lower midline diastasis using paramedian plication stitches. The two V-Loc sutures overlapped by approximately 25% at the midpoint before reversing direction to lock them in place. Next, the residual flank diastasis was plicated with 0 V-Loc sutures.

A 20×25 cm Parietex composite mesh was inserted, and the left flank edge was sutured with overlap onto the previous mesh. An additional 2-0 V-Loc suture was placed along the long edges and the superior and inferior portions of the mesh. An AbsorbaTack tacker was used to flatten the midpoint of the mesh, facilitating fixation of the upper right edge, which was supplemented with tacks and transfascial sutures placed using a suture passer and Vicryl suture.

Final inspection confirmed good edge apposition to the abdominal wall. Hemostasis was achieved, and local anesthetic was injected bilaterally to provide a transversus abdominis plane (TAP) block. The patient tolerated the procedure well, with no complications.

Six months postdischarge, the patient returned to the clinic for follow-up and remained asymptomatic. The first recurrent hernia repair allowed the reconstructed abdominal wall to gradually accommodate the loss of tissue. The second robotic hernia repair addressed the remaining areas of functional deficit and weakness. A timeline summarizing the procedures and services involved in the patient's care is provided in Table 1.

**Table 1:** Timeline of operations

Date	Event	Defect/operation	Mesh
6/2018	Penetrating trauma	20 cm left abdominal wall defect, debridement, sigmoid resection, ABThera	None
7/2018	Staged operations	Washout, colon anastomosis, partial fascial closure	None
7/2018	Abdominal wall coverage	Intraperitoneal bridging repair	20×40 cm Strattice underlay
2/2019	Symptomatic flank hernia	Open fascial approximation	30×30 cm Parietex onlay
8/2019	Recurrent bulge	First robotic plication and repair	20×25 cm Parietex
2/2020	Recurrent defects with diastasis	Second robotic plication and repair	20×25 cm Parietex composite
8/2020	Follow-up	Asymptomatic, no clinical recurrence	None

## Discussion

Flank hernias are rare abdominal wall hernias that range from asymptomatic to symptomatically debilitating, with the potential for life-threatening incarceration and strangulation of visceral organs. Traumatic flank hernias are even rarer, and there is a paucity of published data on their management. This discussion analyzes the presented case, reviews the current literature on traumatic flank hernias, highlights how this case differs from previously reported cases, and identifies opportunities for future research.

The operative course in the presented case demonstrates the use of both open and robotic approaches to manage a recurrent traumatic flank hernia. The ability to use multiple surgical strategies underscores the importance of specialized surgical expertise in managing complex abdominal wall reconstruction and optimizing patient outcomes. This case also contributes to the limited literature on robotic repair of recurrent traumatic flank hernias, making it one of the few documented cases.

There is no gold standard for flank hernia repair. Repair may be performed using open, laparoscopic, or robotic approaches with either extraperitoneal or intraperitoneal mesh placement [5]. The patient initially underwent an open mesh repair that ultimately recurred. He subsequently underwent two robotic repairs and has had no recurrence to date. Although data remain limited, robotic repair appears to be a feasible option for complex abdominal wall reconstruction. Likewise, despite the lack of high-quality comparative data, mesh reinforcement appears to reduce recurrence rates. Although multiple acceptable planes for mesh placement exist, there is currently no evidence demonstrating the superiority of preperitoneal over extraperitoneal repair.

The degree of muscular disruption at the patient's initial presentation is important to recognize. The left abdominal wall was completely disrupted and resembled the reconstructive complexity encountered following abdominal wall sarcoma resection. Although not required in this case, adjunctive techniques such as preoperative botulinum toxin injections to relax the abdominal wall musculature or fascial retention sutures to minimize retraction could have been considered to facilitate delayed reconstruction.

The surgical strategy used in this case aligns with current recommendations regarding mesh use for traumatic abdominal wall defects (TAWDs). The two largest reviews of treatment strategies were conducted by Liasis et al., who included 248 TAWD cases, and Karhof et al., who reviewed 229 cases. Liasis et al. concluded that both the timing of repair and the decision to use mesh should be individualized according to the clinical scenario while recommending mesh reinforcement for patients undergoing delayed repair [6]. Similarly, Karhof et al. found no statistically significant benefit of mesh reinforcement in their pooled analysis, although multiple individual studies demonstrated lower recurrence rates with mesh use [7].

An overview of 17 studies summarized in Table 2 highlights current trends in traumatic flank hernia repair [3,5,8-22]. Computed tomography was the most commonly used imaging modality, although abdominal ultrasonography and magnetic resonance imaging have also been reported [10-22]. Open repair was performed in seven studies, laparoscopic repair in six, combined open and laparoscopic repair in one, and three studies reported a combination of open and minimally invasive approaches. Recurrence was reported inconsistently, and incomplete follow-up, heterogeneous outcome definitions, and potential cohort overlap precluded a meaningful pooled estimate. Reported complications included seroma, hematoma, superficial wound infection, mesh infection, skin necrosis, colocutaneous fistula, and anastomotic leak [5,8,11,18,20,22].

**Table 2:** Overview of 17 studies addressing traumatic flank hernia repair NR, not recorded; SSI, surgical site infection

Study	Surgical technique	Repair technique	No. of patients	Mean defect size (cm^2^)	Mean follow-up (mo)	Recurrence (%)	Complication (%)
Fernandez et al. [9]	Open	Mesh	2	NR	NR	0	0
Harrell et al. [5]	Varied	Mesh vs. primary repair	157	NR	NR	13.7% mesh vs. 10.5% non-mesh	SSI: 25.5% mesh vs. 9.5% non-mesh
Abdelali et al. [10]	Open	Biologic mesh inlay and synthetic mesh	1	NR	NR	0	0
Malkoc et al. [11]	Open	Mesh inlay	1	NR	NR	NR	NR
Lee et al. [12]	Open	Mesh strip suture repair	1	64.96	12	0	0
Heo et al. [13]	Dual (open + laparoscopic)	Mesh	1	70	6	0	0
Azimi-Ghomi et al. [14]	Open	Mesh	1	NR	NR	0	0
Harrell et al. [2]	Open or minimally invasive; early or delayed	Primary repair±mesh	176 repaired (281 total; 86 flank)	NR	NR	11.5% after early repair vs. 15.8% after delayed repair	NR
D'Elia et al. [15]	Open	Primary tissue closure	2	NR	NR	NR	NR
Novitsky et al. [16]	Laparoscopy	Defect closure with mesh underlay and selective bone-anchor fixation	14	NR	35	0	0
Ferris et al. [17]	Laparoscopy	Bridging intraperitoneal Parietex mesh	1	72	36	0	0
Siow et al. [18]	Laparoscopy	Primary closure with Prolene mesh reinforcement	1	NR	NR	NR	0
Honaker et al. [8]	Varied	Primary repair±mesh	30 repaired (38 total)	NR	NR	6.7	NR
Nell et al. [19]	Delayed laparoscopy	Mesh	1	NR	NR	NR	NR
Yadav et al. [20]	Delayed open	Mesh	1	1350 (30x45 cm)	4	0	0
Wilson et al. [21]	Laparoscopy	Mesh	1	NR	12	0	0
Bucher et al. [22]	Single-port laparoscopic	Intraperitoneal composite mesh with tacks and transfascial sutures	52 (55 hernias)	16.2 primary; 48.3 incisional	16 (median)	0	3.8

Timing of traumatic flank hernia repair remains controversial. A multicenter study by Harrell et al. reported no significant difference in recurrence (11.5% vs. 15.8%, P=0.869) or complication rates between early and delayed repair [3]. In contrast, Honaker et al. reported no recurrence (0% vs. 8.3%) and no complications (0% vs. 15%) among patients undergoing delayed repair compared with those undergoing early repair [8]. In our case, surgical repair was intentionally delayed until the patient became symptomatic. This approach is consistent with previous studies recommending delayed repair in patients with life-threatening associated injuries, hemodynamic instability, or extensive contamination [13,17,22].

From the broader perspective of abdominal wall reconstruction, definitive repair using advanced reconstructive techniques should generally be deferred until the acute injury, contamination, and inflammatory response have resolved. During the initial operation, the priorities should be stabilization of the patient, preservation of abdominal wall domain, and prevention of progressive fascial retraction rather than definitive reconstruction. In our patient, the use of Strattice during the initial repair represented an appropriate strategy in the contaminated setting, allowing restoration of abdominal wall continuity while minimizing the risk and consequences of prosthetic mesh infection. Equally important, fascial approximation was achieved whenever feasible during each operation. Maintaining fascial continuity prevented progressive retraction and the formation of increasingly large fibrotic defects that would have made later reconstruction substantially more difficult.

Novitsky et al. demonstrated low recurrence rates with mesh use in both open and laparoscopic flank hernia repair [9,10,12-14,16-21]. In the emergent setting, specialized hernia surgeons advocate a staged approach, initially focusing on temporary repair until acute injuries and inflammation have resolved. Advanced reconstructive techniques, including component separation, should generally be avoided during the acute phase, while prevention of fascial retraction remains a critical goal. Definitive reconstruction should emphasize restoration of core abdominal wall function using durable mesh reinforcement with generous overlap. Although there remains no consensus regarding the optimal plane of mesh placement in traumatic flank hernias, intraperitoneal, retrofascial, and preperitoneal mesh repairs have all demonstrated favorable outcomes with low recurrence rates when combined with primary fascial closure and adequate mesh overlap [23].

An additional challenge in this patient was the associated post-traumatic diastasis and disruption of muscular continuity. Diastasis contributes not only to abdominal wall bulging but also to pain and impaired core function. Flank hernias are particularly challenging because successful reconstruction requires restoration of continuity between the oblique and rectus musculature and their fixed skeletal attachments to the ribs, pelvis, and spine. In this case, closure of the fascial defect together with plication of the associated diastasis allowed the abdominal wall to be re-tensioned as a functional unit rather than simply covering the defect with mesh. By restoring this musculoskeletal continuity, the reconstruction addressed both structural integrity and abdominal wall function, allowing the patient to return to regular exercise and an active lifestyle without pain or recurrent hernia.

One of the primary limitations of this case report is that it describes a single patient, making it difficult to generalize the findings. The unique nature of the penetrating injury and the extensive surgical history may not reflect typical cases of traumatic flank hernias, limiting the applicability of this approach to other patients. Although the multiple reconstructive operations aimed to restore abdominal wall integrity and function, assessment of long-term quality-of-life outcomes remains important. Future studies should include longitudinal follow-up to evaluate patient-reported quality of life and better characterize recurrence after reconstruction. Given the complexity of these repairs, patients at high risk for recurrence should ideally undergo surveillance for at least two years, as many abdominal wall surgeons believe that follow-up at 30 days, three months, or even one year is insufficient to identify late recurrences.

This case contributes to the limited body of literature on traumatic flank hernia repair and uniquely highlights successful robotic intervention in a previously reconstructed abdominal wall. In patients with delayed presentation, recurrent hernia, and prior mesh placement, robot-assisted repair is a feasible and effective option for complex abdominal wall reconstruction. Careful staging of reconstruction, preservation of fascial continuity during the acute phase, restoration of abdominal wall function, and thoughtful long-term surveillance may all contribute to durable outcomes in these challenging patients.

## Conclusions

In the emergency setting, management focused on stabilization, debridement, and temporary restoration of abdominal wall continuity rather than definitive reconstruction. In this patient, a staged strategy using an initial bridging mesh followed by delayed fascial approximation and onlay mesh reinforcement was selected based on the degree of contamination, tissue loss, and available fixation planes. The choice of mesh material and reconstructive approach should be individualized according to the degree of contamination, tissue quality, defect anatomy, available fixation planes, and surgeon expertise.

The ultimate goal is to restore a functional abdominal wall that provides adequate core support. This requires reestablishing continuity between the abdominal wall muscles whenever possible. In this case, the eventration was plicated and secured to the remaining fascial edges, restoring continuity of the oblique musculature despite significant tissue loss. Mesh reinforcement was important because the repair would otherwise have been at high risk for recurrence. When there is substantial loss of flank or rectus muscle tissue, a staged approach allows the initial repair to mature before definitive reconstruction.
